# Supplementary material for: Direct Photochemical Synthesis of Substituted Benzo[b]fluorenes
Source: Org Lett. 2024 Nov 25;26(48):10364–8. doi: 10.1021/acs.orglett.4c03978 (PMC11629382; doi:10.1021/acs.orglett.4c03978)
Supplement: Supplementary file 2 — ol4c03978_si_002.zip [file ol4c03978_si_002.zip › FID for Publication/6a/20240814_RC-2-C-6-141B_01/plots/RC-2-C-6-141B_CARBON_20240814_01_plot01.pdf]

RC-2-C-6-141B

Location **16** | Pulse sequence **CARBON** | Temperature **25** | Suboperator **RuairiCrawford**
Date collected **2024-08-14** | Solvent **acetone** | Spectrometer **localhost.localdomain-vnmrs500** | Operator **MBaumann**

SAMPLE

date	Aug 14 2024
solvent	acetone
file	/home/data/MBaumann/cscb500/RuairiCrawford/2024/20240814_RC-2-C-6-141B_01/RC-2-C-6-141B_CARBON_20240814_01.fid

ACQUISITION

sw	31250.0
at	1.049
np	65536
fb	17000
bs	8
ss	128
d1	1.000
nt	2000
ct	2000

TRANSMITTER

tn	C13
sfrq	125.669
tof	1913.4
tpwr	58
pw	7.700

DECOUPLER

dn	H1
dof	0
dm	yyy
decwave	w
dpwr	36
dmf	13423

PRESATURATION

satmode	n
wet	n

SPECIAL

temp	25.0
gain	50
spin	0
hst	0.008
pw90	15.400
alfa	10.000

FLAGS

il	n
in	n
dp	y
hs	nn

PROCESSING

lb	0.50
fn	not used

DISPLAY

sp	-1802.9
wp	31250.0
rfl	1802.9
rfp	0
rp	-127.1
lp	0

PLOT

wc	169
sc	8
vs	2819
th	2
ai cdc ph	

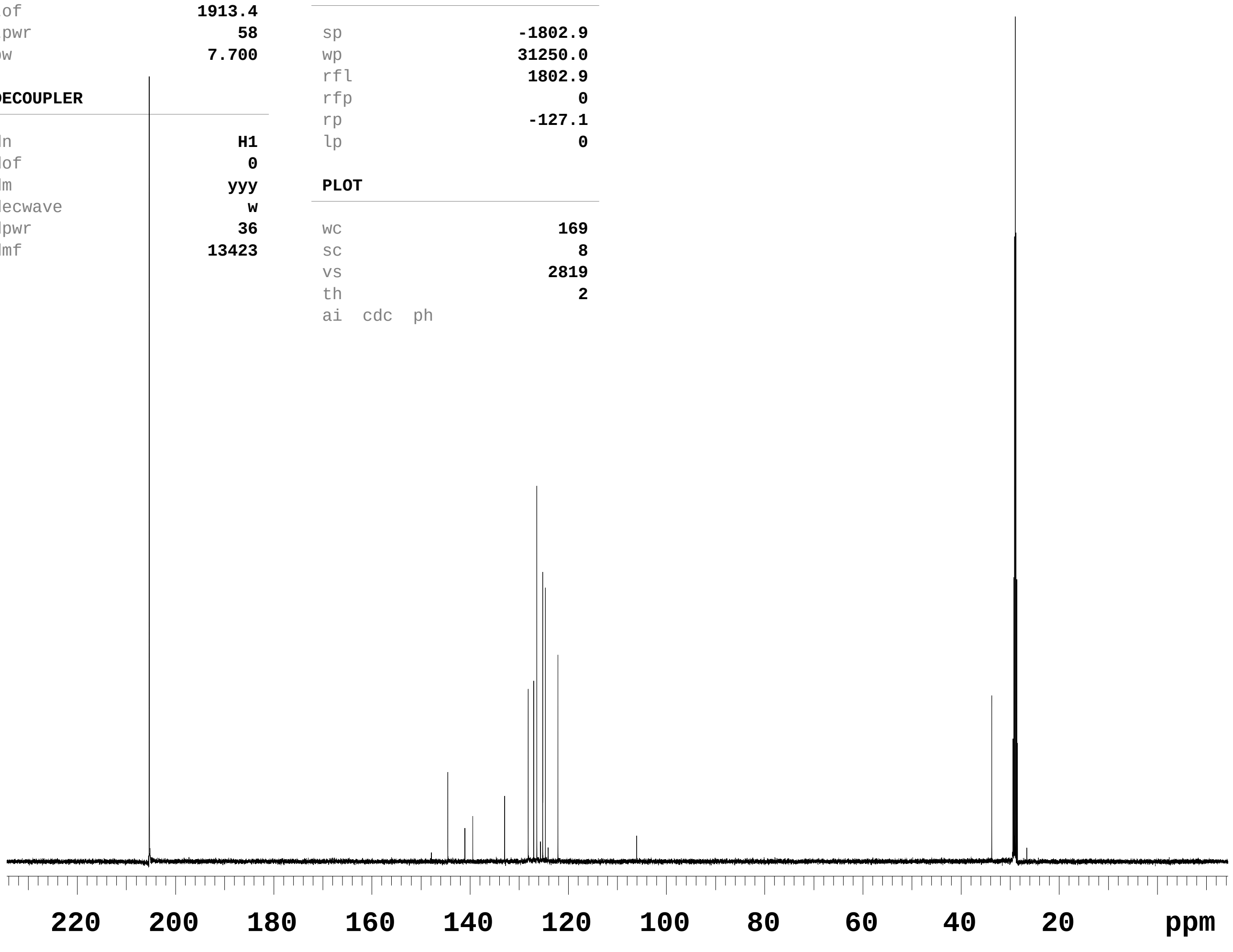

Plotname: RC-2-C-6-141B_CARBON_20240814_01_plot01

Data file /home/data/MBaumann/cscb500/RuairiCrawford/2024/20240814_RC-2-C-6-141B_01/RC-2-C-6-141B_CARBON_20240814_01.fid

Plot date 2024-08-14
